# Heparins Sourced From Bovine and Porcine Mucosa Gain Exclusive Monographs in the Brazilian Pharmacopeia

**DOI:** 10.3389/fmed.2019.00016

**Published:** 2019-02-05

**Authors:** Eduardo Vilanova, Bruno C. Vairo, Stephan-Nicollas M. C. G. Oliveira, Bianca F. Glauser, Nina V. Capillé, Gustavo R. C. Santos, Ana M. F. Tovar, Mariana S. Pereira, Paulo A. S. Mourão

**Affiliations:** Laboratório de Tecido Conjuntivo, Hospital Universitário Clementino Fraga Filho and Instituto de Bioquímica Médica Leopoldo de Meis, Universidade Federal do Rio de Janeiro, Rio de Janeiro, Brazil

**Keywords:** unfractionated heparin, low molecular weight heparin, anticoagulant drugs, antithrombotic drugs, extracorporeal circulation, cardiovascular surgeries, drug regulation, bioequivalence

## Abstract

Most of the unfractionated heparin (UFH) consumed worldwide is manufactured using porcine mucosa as raw material (HPI); however, some countries also employ products sourced from bovine mucosa (HBI) as interchangeable versions of the gold standard HPI. Although accounted as a single UFH, HBI, and HPI have differing anticoagulant activities (~100 and 200 IU mg^−1^, respectively) because of their compositional dissimilarities. The concomitant use of HBI and HPI in Brazil had already provoked serious bleeding incidents, which led to the withdrawal of HBI products in 2009. In 2010, the Brazilian Pharmacopeia (BP) formed a special committee to develop two complementary monographs approaching HBI and HPI separately, as distinct active pharmaceutical ingredients (APIs). The committee has rapidly agreed on requirements concerning the composition and presence of contaminants based on nuclear magnetic resonance and anion-exchange chromatography. On the other hand, consensus on the anticoagulant activity of HBI was the subject of long and intense discussions. Nevertheless, the committee has ultimately agreed to recommend minimum anti-FIIa activities of 100 IU mg^−1^ for HBI and 180 IU mg^−1^ for HPI. Upon the approval by the Brazilian Health Authority (ANVISA), the BP published the new monographs for HPI and HBI APIs in 2016 and 2017, respectively. These pioneer monographs represent a pivotal step toward the safest use of HBI and HPI as interchangeable anticoagulants and serve as a valuable template for the reformulation of pharmacopeias of other countries willing to introduce HBI.

## Essentials

Unfractionated heparin (UFH) is a systemic anticoagulant indispensable for patients undergoing procedures involving extracorporeal circulation such as cardiovascular surgeries and renal-dialysis, with no surrogates approved for use or undergoing clinical trials at the moment (1). Despite the efforts made toward developing synthetic versions, the production of UFH on an industrial-scale still relies on the extraction of crude materials from mammalian tissues through chemical or enzymatic proteolyses followed by purifications with quaternary ammonium salts or anionic-exchange resins and solvent precipitations (2).

UFH was discovered in dog's liver in the early 20th century and afterwards mass-produced using bovine lung as raw material (3). However, bovine-lung UFH was gradually replaced by enhanced formulations sourced from porcine intestinal mucosa (HPI), up to its discontinuation in the 1990s (4). Currently, all the UFH consumed worldwide is sourced from porcine-mucosa, except for some countries, such as Brazil (currently discontinued), Argentina, India, and a handful of Islamic nations, which employ UFH from bovine intestinal mucosa (HBI) concomitantly with the gold-standard HPI (5).

Although clinically employed as interchangeable UFHs and considered as a single pharmaceutical compound (Heparin Sodium) by regulatory agencies, HBI and HPI have contrasting anticoagulant potencies due to their compositional differences (6–10). In this review, we summarize the major scientific, medical, and regulatory episodes leading to the development of the novel compendial monographs published by the Brazilian Pharmacopeia (BP), which consider heparins sourced from porcine or bovine mucosa as distinct active pharmaceutical ingredients (APIs).

## THE Starting-Point

Since 2002, our research group has been performing systematic quality analyses of most of the UFH products available in the Brazilian market (6–11). Among these assessments, we highlight measurements of anticoagulant potencies with clotting (APTT) and chromogenic (anti-FIIa and anti-FXa) assays, evaluations of molecular-weight and presence of contaminants with size-exclusion and anion-exchange chromatography (HPLC) and determinations of disaccharide compositions with nuclear magnetic resonance spectroscopy (NMR). Since the beginning of our partnership with the Brazilian pharmaceutical companies, we have carried out both the anticoagulant and HPLC assays recommended by the BP exclusively with the infrastructure available in our own laboratory. Otherwise, the massive demand of NMR spectra for assessing the chemical compositions of hundreds of batches of UFH has been met thanks to the establishment of a large and well-equipped NMR facility at our institution.

**Figure 1:**
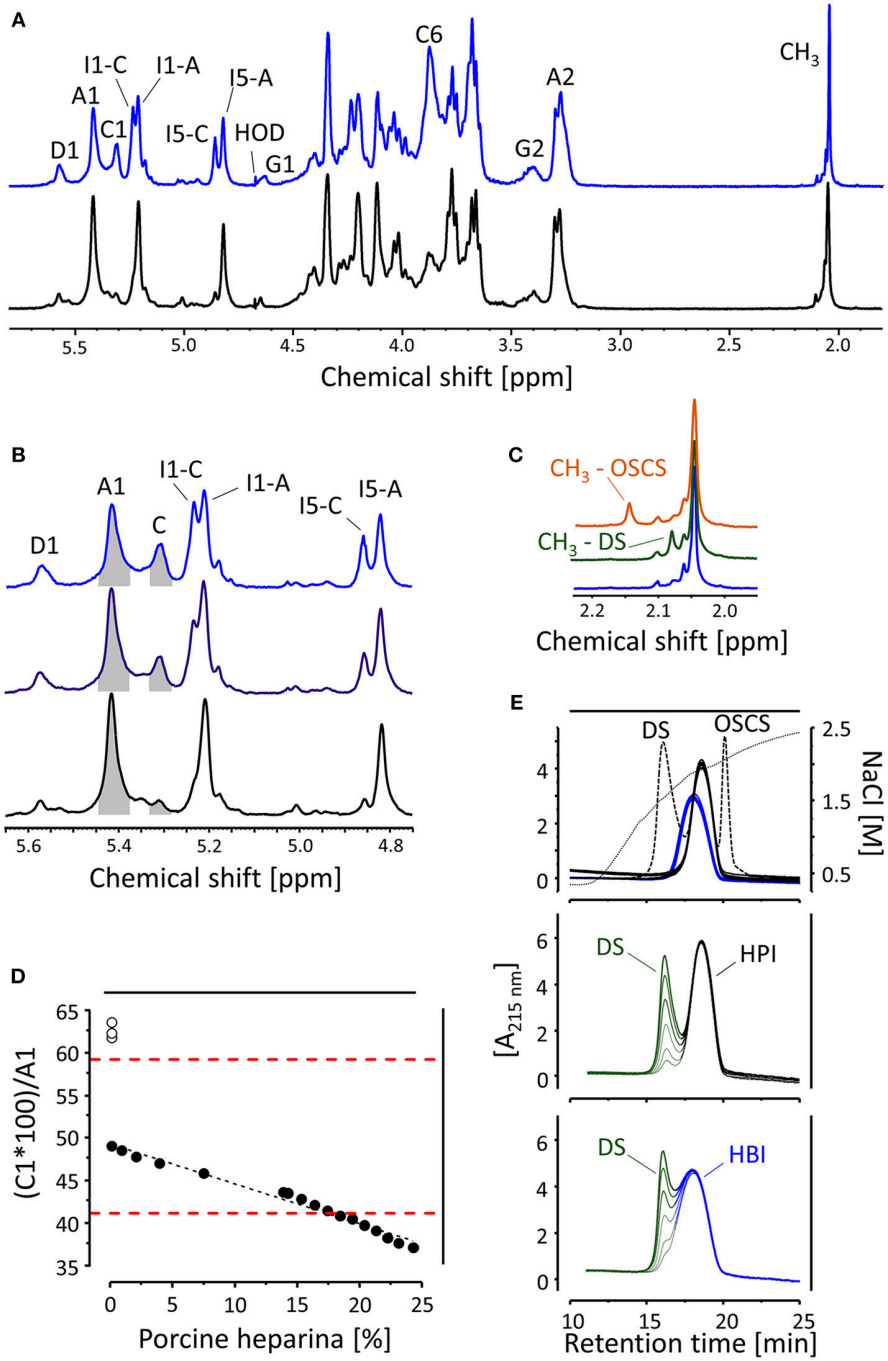
Physical-chemical features of HBI and HPI. **(A)** 1D ^1^H NMR spectra of HBI (in blue) and HPI (in black) showing their characteristic signals, including the fingerprinting A1 and C1, ascribed to the anomeric protons (H1) of *N*,6-disulfated and *N*-sulfated α-glucosamine units, respectively, and H1 and H5 of their respective 2-sulfated α-iduronic acid units (I1-A, I5-A and I1-C, I5-C). For information on the other signals check the section “Establishing chemical differences” and references (6–10, 13, 14). **(B)** Areas of the signals A1 and C1 (in gray) used to calculate the proportions of their correspondent disaccharides in HBI (in blue), HPI (in black) and mixtures (50% of each, in purple). **(C)** 1D ^1^H spectra magnified in the region of the CH_3_ signals of HBI (in blue) and HBI supplemented with DS (in green) or OSCS (in orange). **(D)** *N* values of HBI mixed with increasing quantities of HPI (closed circles) and crude-HBI preparations (open circles); the values outlined by the red dashed lines represent the range recommended for HBI APIs. **(E)** Anion exchange HPLC assessments are effective in detecting DS and OSCS (peaks represented by the continuous dashed line) in preparations of HBI and HPI (several batches of each, in blue and black, respectively) though the partial overlapping of DS (increasing quantities, in green) with HBI (in blue) but not HPI (in black) peaks. The spectra and graphs depicted in the panels are based on results previously published (6–11, 13, 15).

In the early 2000s, five UFH products manufactured by different pharmaceutical companies were available for clinical use as interchangeable anticoagulants in Brazil. Surprisingly, the 1D ^1^H NMR spectra of part of these UFHs revealed unexpected compositional differences. Three of them had spectra similar to that of the 5th International Heparin Standard from NBISC (National Institute for Biological Standards and Control–U.K.), sourced from porcine-mucosa (HPI), in effect at that time, while the other two presented spectra similar to one another but bearing striking differences in the proportions of some fingerprinting signals (11, 12). The major structural difference seen in the conflicting spectra of those two UFHs certainly was an extensive 6-desulfation of their α-glucosamine units, as shown by the 1D ^1^H NMR spectra depicted in the Figure 1A.

We first hypothesized that such 6-desulfation of the α-glucosamine units could be related to inadequate manufacturing processes (11). This explanation seemed plausible at that time because the sulfate-ester linked to the position 6 of the α-glucosamine is the most susceptible to solvolysis (16). However, in-depth analyses of further 1D and 2D NMR spectra allowed us to conclude that the real cause behind those structural differences was the raw material (porcine- or bovine-mucosa) employed to produce the UFHs (6–10). Independent investigations conducted by an Italian group had already demonstrated that HBI contained an increased proportion of 6-desulfated α-glucosamine, thereby strengthening our observation (17–19).

Although none of those UFH products had information on their animal sources available, we were able to ascertain the bovine-mucosa origin of the two brands with higher proportions of 6-desulfated α-glucosamine by comparing their 1D ^1^H NMR spectra with those of other HBI preparations whose animal source was ensured by the manufacturer (6). This finding allowed us to postulate that HBI and HPI have distinct disaccharide compositions and thus their status as the same pharmaceutical compound should be revisited (9).

## Establishing Chemical Differences

Thereafter, we have confirmed such structural differences by analyzing with state-of-the-art NMR techniques the compositions of approximately 500 batches of HPI and 400 batches of HBI manufactured, formulated and/or distributed by different Brazilian pharmaceutical companies (9, 10). The analysis of 1D ^1^H NMR spectra of such a massive number of pharmaceutical preparations has revealed a remarkable batch-to-batch consistency for HBI and HPI and attested to their different disaccharide compositions (9). Further compositional information, obtained from 2D ^1^H/^1^H (COSY, TOCSY, and NOESY) and ^1^H/^13^C (HSQC) NMR spectra and SAX-HPLC analyses of disaccharides released by heparitinase degradation, allowed us to establish the chemical differences between HBI and HPI in fine detail (8, 9).

The major structural difference between HBI and HPI lies in the proportion of disaccharides containing *N*-sulfated α-glucosamine devoid of the sulfate-ester linked to position 6 (6–9, 17–19). HBI has a high proportion of these disaccharides, as easily seen on the 1D ^1^H NMR spectra by the presence of prominent signals C1 and C6, ascribed to the protons H1 (anomeric) and H6 of *N*-sulfated α-glucosamine, and the slightly downfield-shifted H1 and H5 (I1-C and I5-C) of its neighbor 2-sulfated α-iduronic acid (Figure 1A). On the other hand, disaccharides composed of *N,6*-disulfated α-glucosamine (A1) linked to 2-sulfated α-iduronic acid (I1-A and I5-A) are preponderant in HPI (Figure 1A). Besides these fingerprinting signals, we have also observed on HBI spectra increased intensities of the signal D1, ascribed to H1 of *N*-sulfated α-glucosamine linked to β-glucuronic acid, and a reduction of the signal at 5.01 ppm from non-sulfated α-iduronic acid units (Figure 1A) (6–9).

Subsequently, we investigated these structural differences in more detail through a precise quantification of the monosaccharide components of HBI and HPI by integrating their respective signals on 2D ^13^C/^1^H HSQC spectra (9). Disaccharides composed of *N*-sulfated α-glucosamine linked to 2-sulfated α-iduronic acid are abundant in HBI but found reduced in HPI (~28 and 3%, respectively), while those containing *N*,6-disulfated α-glucosamine linked to 2-sulfated α-iduronic acid are present in higher proportion in HPI than in HBI (~68 and 48%, respectively) (9). The HSQC spectra have also revealed that HBI has a diminished proportion of the disaccharide *N*,3,6-trisulfated α-glucosamine linked at the non-reducing side to β-glucuronic acid, which is a pivotal component of the pentasaccharide sequence involved in the binding of heparin to antithrombin (9, 20). Although such a considerable set of structural differences by itself should be enough to raise questions whether HBI and HPI could be considered as similar compounds according to the current standards of bioequivalence (21), it is also the cause of their contrasting anticoagulant activities (9).

## Time OF Crisis

In 2008, HPI products, formulated using APIs manufactured in China that were adulterated with oversulfated chondroitin sulfate (OSCS), provoked 81 deaths and 785 reports of serious adverse events featuring allergic reactions and acute hypotension triggered by OSCS-activation of prekallikrein into kallikrein (22). Medical incidents related to the use of these adulterated HPIs in Brazil were scarce and uncertain despite the presence of OSCS in commercial preparations available for clinical use at that time, including in some employed in our own hospital. We had promptly detected the OSCS on 1D ^1^H NMR spectra (signal at 2.16 ppm) of the preparations and confirmed its presence with agarose-gel electrophoresis analyses. Nevertheless, none of the patients heparinized with the adulterated HPIs presented allergic reactions or acute hypotension during or after procedures conducted in our hospital. Besides the small amounts of OSCS present in the preparations (~5% of the mass), further *in vitro* assays have also revealed that the OSCS contaminant was unable to activate prekallikrein and, for this reason, did not cause hypotension in the patients (23). Considering that OSCSs with a degree of sulfation higher than 3 are readily distinguishable on 1D ^1^H NMR spectra (24), we assumed that such an unusual loss of activity might relate to a discrete desulfation during the transportation and/or shelf-time of the adulterated HPIs made available in Brazil.

Another impacting event that also took place in 2008 was the withdrawal of the traditional HPI brand Liquemine™ (Roche) from the Brazilian market and its replacement by different products formulated with HBI or HPI but labeled as the same UFH (11). Besides being the market leader, Liquemine™ also was the only brand employed in cardiovascular surgeries that required high doses of UFH (11). Shortly after the Liquemine™ discontinuation, several bleeding incidents during or after cardiovascular surgeries began to be reported in Brazilian hospitals (25, 26). At first, the medical community attributed the incidents to the adulterated HPIs though their reported adverse effect was hypotension but not bleeding (22–24). In fact, those bleedings must have been caused by inadequate protamine-neutralizations of HBI formulations administered to the patients due to the use of protocols designed to neutralize HPI (10).

Administration of incorrect doses of protamine to neutralize UFH could provoke bleeding due to: (1) UFH remaining active in the circulation (insufficient doses); (2) the “heparin-rebound” effect; and (3) anticoagulant effect of protamine itself (excessive doses) (27). The higher quantity of HBI (about 2-fold) necessary to achieve the same anticoagulant activity (expressed as Heparin International Units—IUs) of HPI, entails the use of a higher dose of protamine because of the mass-mass nature of this neutralization reaction (10, 15). Such serious medical incidents revealed the risk associated to the simultaneous use of HBI and HPI preparations labeled as a single UFH (Heparin Sodium), with the same anticoagulant activity (IUs) per vial, but containing different quantities of APIs nevertheless (10).

In 2009, the Heparin Sodium monograph of the BP underwent an extensive revision for incorporation of new requirements and recommendations adopted by reference pharmacopeias [e.g., (13, 14)]. One of these changes was the adjustment of the minimum anticoagulant activity from 140 to 180 IU mg^−1^ to meet the average potency of the HPI products available in the market (28). When it became clear that HBI API's would never reach such an anticoagulant potency, the last HBI product still available for clinical use was withdrawn and since then only two HPI products, both imported from China, have been sold in Brazil (9, 10). However, some Brazilian pharmaceutical companies were willing to pursue the reintroduction of HBI notwithstanding its inadequacy to the UFH monograph in effect at that time.

## The New Monographs

The efforts toward the reintroduction of HBI in Brazil began in 2010, when the BP promoted the formation of a special committee, composed by ourselves and other Brazilian authorities on the research, regulation, production, and medical use of UFH, to implement new monographs considering HBI and HPI as distinct pharmaceutical compounds. Initially, the committee proposed the substitution of the sole UFH monograph in effect at that time by two complementary monographs approaching HBI and HPI APIs (UFH powders) separately and a third monograph for the UFH final products (injectable solutions) formulated with either APIs (29–31).

### Chemical Requirements

Once the strategy for the implementation of the new monographs had been defined, the committee established the specific physical-chemical recommendations for HBI and HPI APIs. We agreed to incorporate compositional requirements based on 1D ^1^H NMR and anion-exchange HPLC assessments in the new monographs (29, 30). Recommendations regarding molecular-weight distribution were not incorporated because of the preliminary nature of the information available on HBI at that time.

The structural requirements (29, 30) included a precise identification (ppm ± 0.03) of the following fingerprinting signals on the 1D ^1^H NMR spectra of the APIs: A1 (5.42 ppm) from H1 of *N,6*-disulfated α-glucosamine; C1 (3.28 ppm) from H1 of *N*-sulfated α-glucosamine; I1 (5.21 ppm) from H1 of 2-sulfated α-iduronic acid and CH_3_ (2.05 ppm) from the methyl group of *N*-acetylated α-glucosamine (Figures 1A,B). It would also recommend the absence of several signals from possible contaminants (29, 30), including one at 2.16 ppm, attributed to CH_3_ of the OSCS (Figure 1C).

After identification, the signals A1 and C1 must be integrated (Figure 1B) and then subjected to the following equation to characterize the amount of 6-*O* desulfation:

$$\begin{array}{l} \frac{\text{C}1\text{ x }100}{\text{A}1}=N \end{array}$$

Where *N* represents the area of C1 relative to A1 and must result in values up to 20 for HPI and within 42–58 for HBI (Figure 1D). A broad range was recommended for HBI in order to cover eventual structural variations of upcoming products but also conservative enough to exclude APIs prepared with non-purified (crude) HBI (open circles, Figure 1D). Besides being a key parameter to attest to the animal source of the APIs, *N* is also useful to identify preparations containing mixtures of HBI and HPI (Figure 1D), which then allowed the committee to stipulate a maximum level of mixture (16%) in the new monographs (29, 30).

Requirements regarding the identification of contamination with other glycosaminoglycans (GAGs), *viz*. dermatan sulfate (DS) and OSCS, were based on anion-exchange chromatography assessments (29, 30). DS and OSCS contaminants present in both HBI and HPI APIs are easily detectable on the chromatograms despite that there is a partial overlapping of HBI and DS peaks (Figure 1E). Our efforts to improve the detection of DS into HBI preparations by testing different anion-exchange columns and elution strategies were unsuccessful thus far, but we are still looking for new alternatives nevertheless. In conclusion, the set of physical-chemical recommendations incorporated in the new monographs for HBI and HPI APIs are robust enough to assure their animal source and to avoid significant GAG-based contaminations.

### Anticoagulant Activities

Although the committee had agreed rapidly on the physical-chemical recommendations to be incorporated into the new monographs, specifications for the anticoagulant activity of the HBI APIs were the subject of long and intense disputes. Most of the discussions have centered on the conflicting anticoagulant potencies attributed to HBI by different groups in previous decades. Besides being a pivotal pharmacological parameter, the determination of the potency of HBI APIs (IU mg^−1^) also has important economic implications because UFH products are commercialized in terms of IUs rather than by weight.

**Figure 2:**
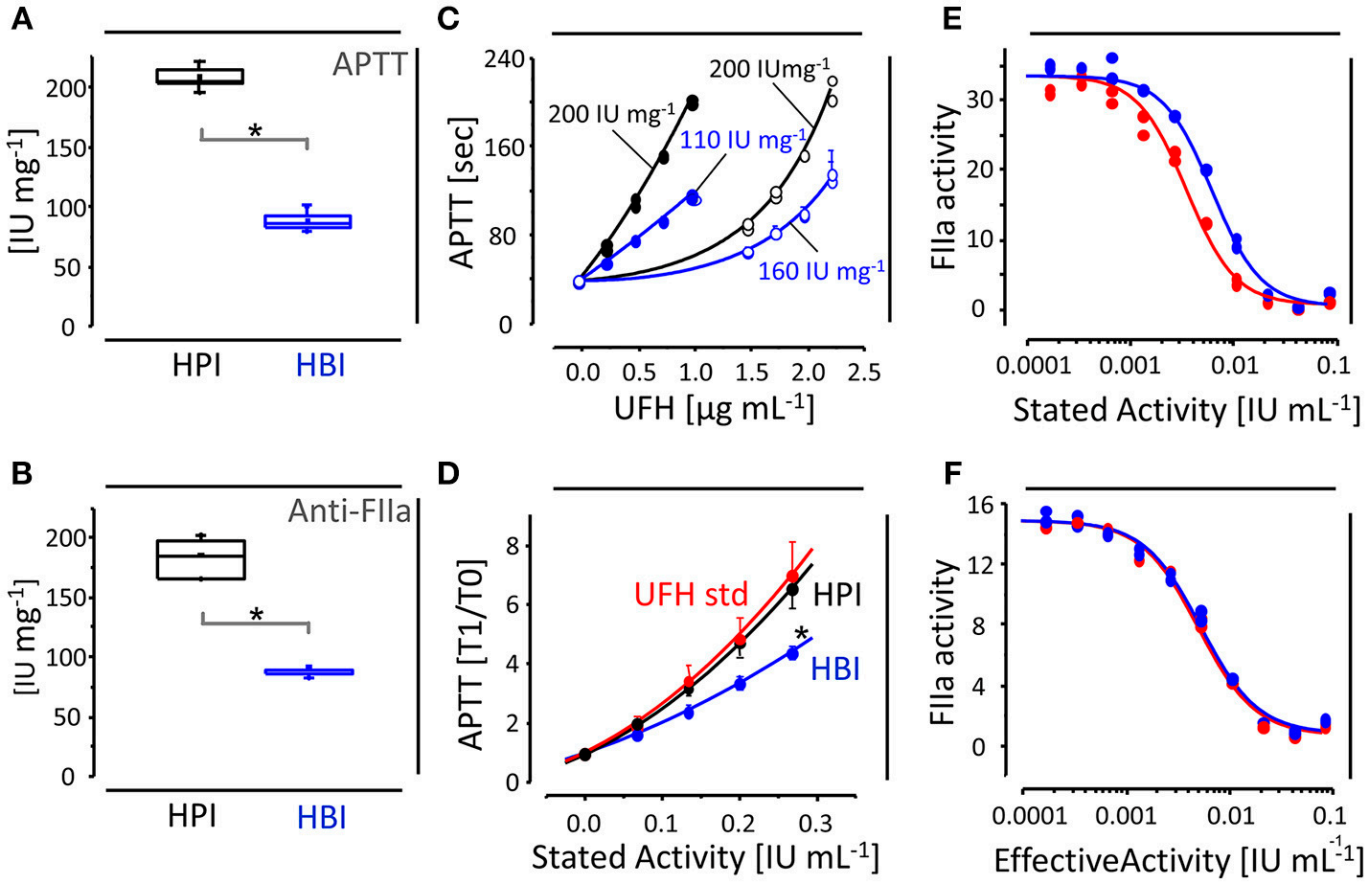
Anticoagulant activities of HBI and HPI. Average anticoagulant activities (IU mg^−1^) of HBI (in blue) and HPI (in black) APIs achieved in APTT **(A)** and anti-FIIa assays **(B)**. **(C)** APTT assays performed with human (closed circles) or ovine (open circles) plasma; note the overestimated potencies achieved by HBI (in blue) but not HPI (in black) in the assays with ovine plasma. **(D)** APTT assays with HPI formulations (in black) based on stated potencies of 180 IU mg^−1^ yield curves coincident to the International Heparin Standard (in red), while HBI final products (in blue) based on overestimated potencies (140–160 IU mg^−1^) achieve potencies significantly lower. Anti-FIIa-based parallel line assays showing curves of the International Heparin Standard (in red) and HBI formulations (in blue) based on the potency stated by the manufacturer **(E)** and effectively determined by us **(F)**. ^*^in the panels indicate significant differences (*p* < 0.05). The graphs depicted in the panels are based on results previously published (6–11, 13, 15).

Our partnerships with Brazilian pharmaceutical companies allowed us to assess the anticoagulant activities of hundreds of batches of HBI and HPI APIs and final products. Most of those batches have had their potencies determined by APTT and/or anti-FIIa assays (9, 10). This extensive set of assessments revealed consistent potencies between the different batches of HBI and HPI and confirmed their different anticoagulant activities. Both anti-FIIa and APTT assays (Figures 2A,B) have shown HBI with half of the potency of HPI (~100 and 200 IU mg^−1^, respectively) (6–9). Such a robust (large number of batches), thorough (state-of-the-art assays), reliable (pharmaceutical-grade preparations) and systematic (standardized) assessment of the anticoagulant activity of HBI is unprecedented.

Nevertheless, the diminished anticoagulant activity of HBI (~100 IU mg^−1^) proposed by our group was not accepted immediately by the committee because of reports of enhanced potencies by other groups. One of the first evaluations (1979) of the anticoagulant activity of HBI showed a French preparation achieving 143 IU mg^−1^ (32). Shortly after, in 1982, a Brazilian group reported an HBI with 157 IU mg^−1^ (33). The increased potencies seen in these early reports could be related to compositional differences between the old and new HBI preparations. However, an HBI British standard prepared by the NIBSC in the early 1960s presents anticoagulant activity (~100 IU mg^−1^) and structure (1D ^1^H NMR spectrum) similar to those of HBI preparations available nowadays (5, 34, 35). Moreover, independent groups have recently reported newly produced HBIs achieving potencies of 142 and 172 IU mg^−1^ (36, 37). Therefore, the conflicting and howsoever increased potencies attributed to HBIs must be related to inconsistencies in the assessments of their anticoagulant activities rather than compositional differences among them.

Several factors can influence the outcomes of anticoagulant assays. The clotting assay APTT certainly is the most susceptible to inconsistencies caused by the use of different plasmas, *viz*. porcine, ovine or human (38). In fact, HBI APIs show overestimated anticoagulant activities (~160 IU mg^−1^) in APTT assays with ovine-plasma (open circles in blue, Figure 2C), while in assays with human-plasma (closed circles in blue, Figure 2C), the potencies (~110 IU mg^−1^) are close to those seen in anti-FIIa assays (~100 IU mg^−1^, Figure 2B). Otherwise, HPI APIs achieve equivalent potency (~200 IU mg^−1^) in both APTT (ovine- or human-plasma) and anti-FIIa assays (Figures 2B and C). Currently, plasma-based methodologies are no longer recommended by most pharmacopeias and hence the requirements on anticoagulant potency/dosage for UFHs rely exclusively on anti-FIIa assessments (13, 14, 28–31).

HBI products available in Brazil up to 2009 were formulated with basis on the overestimated anticoagulant activities determined with ovine-plasma APTTs (140–160 IU mg^−1^), which yield preparations achieving potencies (IU mL^−1^) approximately 30% below the heparin standard curve in human-plasma APTTs (Figure 2D). On the other hand, HPI formulations based on the potencies stated by the manufacturers (~180 IU mg^−1^) present APTTs coincident to the heparin standard (Figure 2D). Moreover, anti-FIIa-based parallel line assays performed with formulations based on the overestimated potencies formerly stated to HBI (140–160 IU mg^−1^) result in shifted anti-FIIa curves (Figure 2E), while those prepared by following the effective potency (~100 IU mg^−1^) present curves coincident to the heparin standard (Figure 2F). In addition to supporting the diminished anticoagulant potency of HBI determined by us and other groups (6–10, 15, 39), these findings also demonstrate that the current heparin standards based on HPI are suitable to evaluate the anticoagulant activity of UFH final products formulated with either APIs, which in turn makes the introduction of new HBI standards unnecessary.

After all, the robust set of evidence outlined above compelled the committee to incorporate requirements on anticoagulant activity/dosage based on the values proposed by our group. According to the acceptance criterion of the new monographs, HBI and HPI APIs must achieve at least 100 and 180 IU mg^−1^, respectively, in anti-FIIa assays and yield anti-FXa/anti-FIIa ratios between 0.9 and 1.1 (29, 30). With these values in hand, we proceeded with the preparation of the monographs. After a final round of adjustments, the new monographs for HPI (*Heparina Sódica Su*í*na)* and HBI (*Heparina Sódica Bov*í*na)* APIs were ultimately approved by the Brazilian Health Authority (ANVISA) and then published in the first (2016) and second (2017) supplements of the 5th edition of the BP (29, 30).

## Final Considerations

The current production of heparin mostly based on porcine-mucosa preparations manufactured in China (more than 60%) may be compromised by: (1) the insufficiency of raw material to meet the increasing demands of UFH and LMWHs and (2) the risk of shortages caused by diseases in the Chinese pig herd (5, 40, 41). Different stakeholders involved in the production, regulation and medical use of UFH have been recommending the introduction of HBI products to reinforce the global supply chain of this life-saving anticoagulant (5). Therefore, two UFH products, with distinct pharmacological features, might soon be simultaneously available worldwide (10). However, the concomitant use of HBI and HPI, disregarding their differing anticoagulant activities, can provoke serious medical incidents, such as those experienced in Brazil (25, 26). The pioneer initiative of the BP in publishing novel monographs approaching separately the distinct pharmacological features of HBI and HPI certainly was a pivotal step toward their safe use as interchangeable UFHs; therefore, such a framework should be used as a template for the reformulation of pharmacopeias of other countries willing to introduce HBI.

## Author Contributions

PM was member of the committee tasked to implement the pharmacopeias. PM, BV, S-NO, BG, GS, AT, NC and MP participated in the development of the pharmacopeias, and EV and PM wrote the review.

### Conflict of Interest Statement

The authors have performed quality analyses of heparins produced by the following Brazilian pharmaceutical companies: Eurofarma, Cristália, Blau Farmacêutica, Hipolabor, and Extrasul.
